# A meta-analysis of the long-term outcomes following surgery or endoscopic therapy for chronic pancreatitis

**DOI:** 10.1007/s00423-022-02468-x

**Published:** 2022-03-22

**Authors:** Daniel Ll Hughes, Ioan Hughes, Michael A. Silva

**Affiliations:** 1 Department of Oncology, University of Oxford, Old Road Campus Research Building, Old Road Campus, Off, Roosevelt Dr, Headington, Oxford, OX3 7DQ UK; 2 Department of Hepatobiliary and Pancreatic Surgery, Oxford University Hospitals NHS Foundation Trust, Oxford, UK; 3 Queen’s University Belfast, University Road, Belfast, Northern Ireland

**Keywords:** Chronic pancreatitis, Surgical management, Endoscopic management, Quality of life, Outcomes

## Abstract

**Purpose:**

Refractory abdominal pain is a cardinal symptom of chronic pancreatitis (CP). Management strategies revolve around pain mitigation and resolution. Emerging evidence from observational studies highlights that surgery may result in superior pain relief when compared to endoscopic therapy; however, its impact on long-term quality of life or functional outcome has yet to be determined.

**Methods:**

A search through MEDLINE, PubMed and Web of Science was performed for RCTs that compared endoscopic treatment with surgery for the management of CP. The main outcome measure was the impact on pain control. Secondary outcome measures were the effect on quality of life and the incidence rate of new onset exocrine and endocrine failure. Data was pooled for analysis using either an odds ratio (OR) or mean difference (MD) with a random effects model.

**Results:**

Three RCTs were included with a total of 267 patients. Meta-analysis demonstrated that operative treatment was associated with a significantly higher rate of complete pain control (37%) when compared to endoscopic therapy (17%) [OR (95% confidence interval (CI)) 2.79 (1.53–5.08), *p* = 0.0008]. No difference was noted in the incidence of new onset endocrine or exocrine failure between treatment strategies.

**Conclusion:**

Surgical management of CP results in a greater extent of complete pain relief during long-term follow-up. Further research is required to evaluate the impact of the time interval between diagnosis and intervention on exocrine function, combined with the effect of early up-front islet auto-transplantation in order to determine whether long-term endocrine function can be achieved.

**Supplementary Information:**

The online version contains supplementary material available at 10.1007/s00423-022-02468-x.

## Introduction

Chronic pancreatitis (CP) is a debilitating disease. Patients suffer from a protracted disease course with serial episodes of recurrent pain. Abdominal pain serves as one of the cardinal hallmarks of CP [1]. The pain can be continuous or intermittent in nature [2, 3]. The specific mechanism of chronic pain in CP is poorly understood and it is likely to be multifactorial in origin [4]. The proposed mechanisms include ductal hypertension, elevated intraparenchymal tissue pressure and local neuronal remodelling with infiltration of inflammatory cells [5–7]. However, one aspect that is clear is the marked morbidity associated with CP. This is evident in the increasing dosing regimens of analgesia required by patients in order to achieve pain control, in addition to the marked use of opiate analgesia and concurrent opiate dependence observed in this cohort of patients [8, 9]. Long-term opiate use may result in opioid-induced hyperalgesia and gut dysmotility which may also contribute to the complexity of the pain [8]. Over time, successive episodes of inflammation lead to the destruction of normal pancreatic parenchyma and its replacement with non-functioning fibrosis which results in the development of both exocrine and endocrine failure [10]. Long-term inflammation of the pancreas results in a marked increased risk of developing pancreatic cancer [11]. The majority of patients with CP are of working age and as a consequence to their disease, they may be unable to integrate or contribute to society. As a result, social segregation may occur.

The management of CP remains a challenge. Historically, surgery was performed in the context of failed medical therapy [12]. Therefore, a marked time delay was implemented between the onset of the disease and definitive treatment, with only patients with advanced and well-established disease being listed for surgery. The nature of surgery is dependent on multifactorial aspects: notably the disease distribution throughout the gland and the size and the morphology of the main pancreatic duct [13, 14]. In general, surgery adopts one of the three different approaches: resection of diseased tissue, ductal drainage and a combination of resection and drainage [13]. All procedures share a common aim, to alleviate chronic pain [13].

Recent advances in endoscopic techniques have resulted in novel approaches to managing CP [15]. Main pancreatic duct strictures can be dilated and stented [16]. Obstructing ductal stones can be removed or fragmented through lithotripsy [17]. These serve as a minimally invasive approach to managing the disease. Small, observational studies have demonstrated a cohort of patients managed successfully with endoscopic therapy without escalation to surgical treatment [18, 19]. It has been proposed by some that endoscopic therapy may serve as a bridging treatment prior to surgery. However, such an approach may inadvertently introduce a time lag; thus, patients will have developed advanced disease at the time of surgery. This time delay results in prolonged opiate use (which may impact on long-term outcomes following surgery with the development of narcotic bowel syndrome and gut dysmotility), in addition to extensive gland fibrosis that may contribute to higher rates of exocrine and endocrine failure over time [20]. An emerging concept from the wider literature is that patients with CP may benefit from early surgery. Based on observational studies, higher rates of long-term pain control are achieved with early surgery [21, 22]. However, these studies lack a direct comparison between surgery and endoscopic therapy. Another important outcome measure that has yet to be determined is whether early surgery improves quality of life and maintains functioning exocrine and endocrine capacity. The aim of this meta-analysis was to review published level 1 evidence, randomised control trials (RCTs) on the topic to determine whether long-term pain control is achieved with surgical management or endoscopic therapy for CP, whether patients’ quality of life was impacted and if exocrine and endocrine pancreatic function was preserved.

## Methods

This systematic review and meta-analysis was performed with adherence to the Preferred Reporting Items for Systematic Reviews and Meta-Analyses (PRISMA) guidance [23]. A broad and extensive search through the concurrent literature regarding the management of chronic pancreatitis was performed. Three online data archives were interrogated (MEDLINE, PubMed and Web of Science) for appropriate studies published between 1946 and July 2020. A specific search criterion was developed prospectively and was subsequently adapted for each individual data archive. The search criterion adopted a PICO search strategy in order to identify appropriate studies: patients with chronic pancreatitis (of all aetiological causes) undergoing definitive treatment with surgery. Endoscopic treatment served as the comparator group. The defined outcomes of interest were pain control, quality of life and both endocrine and exocrine function. A thorough search of the pre-existing literature regarding the management of chronic pancreatitis was performed. The search was conducted through searching through titles and abstracts utilising keywords for each PICO criterion aforementioned. The Boolean operators (AND or OR) were integrated into the search in order to expand maximum article capture. The asterisk character was added to keywords to serve as a truncation operator and to broaden the search strategy to maximise data collection. All search results were integrated into one list and exported into Rayyan software for the removal of duplicate references [24]. The reference lists of all included studies were hand searched in order to identify other potentially relevant studies.

Two independent authors (DH and IH) performed screening of the individual titles and abstracts of the final search strategy against predefined inclusion and exclusion criteria. A full-text screen was subsequently performed. Any discrepancies were resolved by consensus following a discussion. The inclusion criteria consisted of studies that compared endoscopic intervention and surgery in the management of CP. In order to evaluate level 1 evidence as per the Oxford Centre for evidence-based medicine, only randomised control trials were included in this study [25]. Articles were required to have a minimum of 12-month follow-up and to have reported on specific outcome measures (pain control, quality of life, endocrine and exocrine function). The exclusion criteria consisted of non-randomised studies or articles that did not record specific outcome measures. Non-English articles were translated and screened. Conference abstracts and non-full-text articles were excluded.

Data extraction was performed by two independent authors (DH and IH), and the results were compared and discussed. A specific predesigned data collection pro forma was used for data collection. Data regarding the trial design and the demographics of patients included was collected. The nature of the surgical management (procedure performed) and the endoscopic intervention (lithotripsy, stent insertion, sphincterotomy) was noted. Specific outcome measures were extracted and recorded: pain control, Izbicki score, exocrine and endocrine function. The primary outcome measure of this meta-analysis was the impact of either treatment (surgery or endoscopic intervention) on patients’ pain control. The use of a scoring system for pain control was recorded in addition as to whether the Izbicki score was utilised [26]. The Izbicki score is a validated questionnaire that encompasses 4 domains to create a pain score (intensity of pain, frequency of pain, inability to work and analgesia requirements). The score is assessed on a scale, with a score of 100 representing maximum pain. Secondary outcome measures were the incidence of new onset endocrine or exocrine failure post-procedure.

Methodological quality and an assessment of bias for each included article were formally assessed using the Cochrane Collaboration tool for assessing risk of bias (RoB 2.0) [27]. The overall quality of the evidence was evaluated as per the GRADE (Grading Recommendations Assessment, Development, and Evaluation) criteria (supplementary material) [28]. An assessment of publication bias through funnel plot depiction was not possible as less than 10 articles were included.

Data regarding predefined outcomes was summarised, grouped and formally analysed using the Review Manager (RevMan, version 5.4) platform. Subsequent analysis of dichotomous variables was calculated utilising the odds ratio (OR) as the numerical statistic alongside 95% confidence interval (CI) with the Mantel–Haenszel method. Post-procedural pain relief, overall morbidity and mortality after surgery and endoscopy were analysed, alongside exocrine and endocrine dysfunction. Continuous data (such as Izbicki score) was assessed using mean difference (MD) and 95% CI with the inverse variance method. In order to account for clinical heterogeneity, the random effects model was used. Results were tabulated and visualised through forest plots. Assessment of heterogeneity amongst studies was assessed using the *I*^2^ value. *I*^2^ value was considered to represent low (< 25%), middle (25–75%) or high degrees (> 75%) of heterogeneity. Results were deemed to be of statistical significance when *p* < 0.05.

## Results

**Fig. 1:**
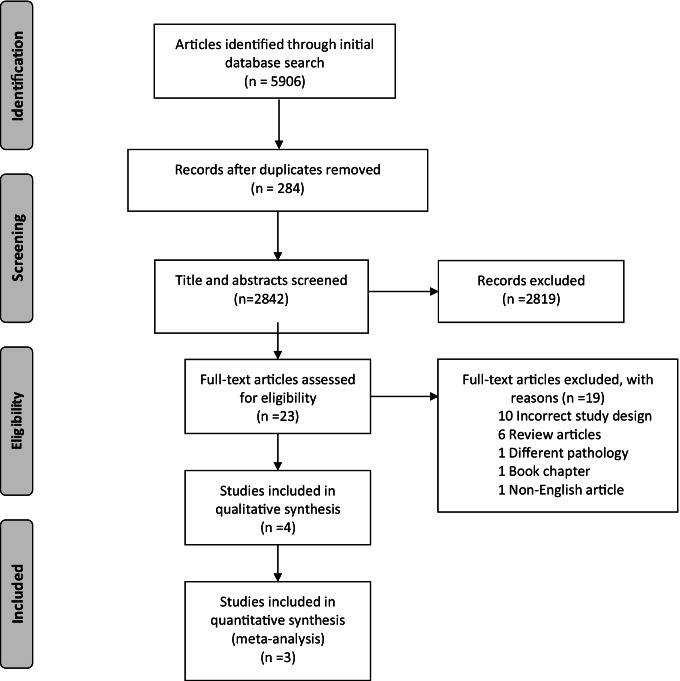
PRISMA flow diagram of included studies

Following a thorough and systematic search through the pre-existing published literature, a total of 5906 articles were identified. After removing duplicate articles and cross-referencing with the predefined study inclusion and exclusion criteria, a total of four articles were included (Fig. 1) [29–32]. These included 3 separate randomised clinical trials (RCTs), one of which had published the results of 5-year follow-up data separately [31]. The trials were published in 2003, 2007 and 2020 respectively and were conducted in European centres (2 from the Netherlands and 1 from the Czech Republic) [29, 30, 32] (Table 1). Both the published studies from Díte et al. and Cahen et al. were monocentric open-label randomised clinical trials [29, 30], whereas the ESCAPE trial, produced by Issa et al., was a multicentre, open-label randomised clinical superiority trial [32]. Recruitment to the trial of Cahen et al. was ended prematurely following an interim analysis that demonstrated a significant difference in outcomes (*p* < 0.001) [30]. All 3 trials included patients with CP of all aetiologies (apart from Issa et al. which excluded patients with autoimmune pancreatitis) [32]. The exclusion criteria of all 3 trials had recurring themes; however, some differences were noted; Cahen et al. excluded patients with pancreatic head enlargement (> 4 cm), whereas Issa et al. excluded patients with prolonged opiate use perioperatively [30, 32]. All three trials reported a primary outcome measure of detecting a clinical change in pain control (2 of which by a reduction in the Izbicki score and the remaining trial by a reduction in the Melzack score) [20, 29, 32]. Two trials reported a 5-year follow-up, and the outstanding trial reported an 18-month follow-up [20, 29, 32].

**Table 1:** Study characteristics

	Díte et al. [29]	Cahen et al. [30]	Issa et al. [32]
Year published	2003	2007, 2011	2020
Journal (impact factor)	Endoscopy (7.34)	Gastroenterology (20.8) NEJM (70.67)	JAMA (45.54)
Country	Czech Republic	Netherlands	Netherlands
Design	RCT*, monocentric, open-label	RCT*, monocentric, open-label	RCST^£^, multicentre (7 sites), open-label
Power calculation used to determine study size	NS^%^	Yes	Yes
Successful recruitment to power calculation study size	NS^%^	No (early termination following interim analysis)	Yes
Randomisation method	NS^%^	Randomisation via varying blocks size (4 or 6) by an automated assignment system that concealed the treatment assignments	Randomisation via varying block size (2, 4, or 6) by an automatic assignment system that concealed allocation
Inclusion criteria	CP diagnosed on imaging. Obstructive CP with dilated duct strictures ± stones in the pancreatic head or body. Painful CP with 3 Melzack’s score > 3. Failure of conservative management for 3 years. Duration of disease > 5 years	CP diagnosed based on clinical symptoms and morphological changes based on imaging Obstruction of the pancreatic duct with proximal duct dilatation. Severe/recurrent pancreatic pain requiring opiates	CP diagnosed on M-ANNHEIM diagnostic criteria with one or more of the following criteria: calcifications or moderate/marked ductal lesions on imaging and/or marked and persistent exocrine insufficiency. Dilated pancreatic duct (> 5 mm). Severe pain requiring opioid analgesia (for 3 days a week for at least 2 consecutive weeks
Exclusion criteria (pancreatitis/disease specific)	Prior interventional therapy for chronic pancreatitis (endoscopic or surgery)	Enlargement of the pancreatic head > 4 cm Contraindications to endoscopic treatment Previous pancreatic surgery	Prolonged use of opioids (weak opioids for > 6/12 or strong opioids > 2/12 in 2 years prior to randomisation) Previous pancreatic surgery or endoscopic therapy to the pancreatic duct. Autoimmune pancreatitis Intraductal stones fully impacting the entire pancreatic duct or exclusively located in pancreatic tail
Primary outcome measure	Clinical change in pain control (reduction in Melzack score)	Clinical change in pain control (reduction in Izbicki score)	Clinical change in pain control (reduction in Izbicki score)
Follow-up duration	5 years	2 and 5 years reported	18 months

^*^Randomised clinical trial, ^£^randomised clinical superiority trial

A total of 267 patients with CP were included amongst the 3 RCTs (Table 2). Díte et al. had the largest study size with 76 patients in the surgery arm and 64 patients in the endoscopy arm respectively [29]. Alcohol-induced CP was the most frequent aetiology for pancreatitis in all three RCTs. The pain pattern of patients was recorded in 2 RCTs, where a continuous pain pattern was most frequently observed [30, 32]. A high prevalence of current smokers was observed in the CP patient cohorts (> 79%) [30, 32]. Pre-existing exocrine insufficiency at baseline was much more prevalent than endocrine insufficiency [30, 32]. Two RCTs reported baseline SF-36 quality of life scores (a standardised questionnaire where a low score denotes greater disability), in both the physical and mental health domains. No mean score greater than 38 was recorded in either trial [30, 32].

**Table 2:** Patient demographics at baseline

Demographics	Díte et al. [29]		Cahen et al. [30]		Issa et al. [32]	
	Surgery (*n* = 76)	Endoscopy (*n* = 64)	Surgery (*n* = 20)	Endoscopy (*n* = 19)	Surgery (*n* = 44)	Endoscopy (*n* = 44)
Mean age (SD)	NS	NS	46 (12)	52 (9)	49 (10)	56 (9)
Male sex (%)	NS	NS	15 (75)	11 (58)	33 (75)	34 (77)
Aetiology of pancreatitis — No. (%)						
Alcoholic	*	*	12 (60)	9 (47)	34 (77)	27 (61)
Idiopathic	NS	NS	5 (25)	7 (37)	7 (16)	12 (27)
Hereditary	NS	NS	1 (5)	1 (5)	1 (2)	1 (2)
Other	NS	NS	2 (10)	2 (11)	2 (5)	4 (9)
Pain pattern — No. (%)						
Continuous	NS	NS	11 (55)	12 (63)	29 (66)	35 (80)
Intermittent	NS	NS	9 (45)	7 (37)	15 (34)	9 (20)
Enlarged pancreatic head — No. (%)	NS	NS	^£^	^£^	21 (48)	23 (52)
Izbicki pain score — mean (SD)	NS	NS	69 (18)	73 (12)	63 (19)	64 (16)
Current smoker — No. (%)	NS	NS	17 (85)	15 (79)	41 (93)	36 (82)
Duration of symptoms (months) — median (IQR)	NS	NS	21^$^	16^$^	12 (3–60)	12 (5–36)
Exocrine insufficiency — No. (%)	NS	NS	16 (80)	13 (68)	33 (75)	34 (77)
Endocrine insufficiency — No. (%)	NS	NS	4 (20)	4 (21)	8(18)	10 (23)
SF-36 quality of life score — mean (SD)						
Physical health scale	NS	NS	35 (8)	31 (8)	35 (7)	31 (8)
Mental health scale	NS	NS	37 (12)	33 (8)	38 (13)	36 (11)

*NS* not specified. *Alcohol-related pancreatitis recorded in 87.8% of the cohort, ^£^exclusion criteria, ^$^data recorded as mean

Endoscopic management was performed in 127 patients across the 3 RCTs (Table 3). Lithotripsy was required in the management of ductal stones in > 75% of the patient cohort in 2 of the trials. High rates of balloon dilatation and pancreatic duct stenting were recorded in both Cahen et al. and Issa et al. 2020 [30, 32]. Complete duct clearance was achieved and reported in 2 RCTs (62% and 89% respectively) [30, 32]. The complication rate following endoscopic management ranged from 8 to 58%. Post-endoscopy pancreatitis was observed in all 3 RCTs. Only one death following endoscopic treatment was recorded across all 3 RCTs. Cahen et al. [30] reported 1 death — a perforated duodenal ulcer 4 days post-lithotripsy. The patient had been receiving concurrent nonsteroidal anti-inflammatory analgesia [30]. Surgical management of CP was performed in 137 patients across the 3 RCTs (Table 4). The nature of the surgical intervention was recorded and presented as resection only, drainage procedure or a combination of resection and drainage. In Díte et al., resection was the most frequently performed surgical procedure for CP (61/76, 80%), of which 33 were duodenum-preserving pancreatic head resections [29]. Both Cahen et al. and Issa et al. reported higher rates of drainage procedures (80 and 55% respectively), of which pancreaticojejunostomy was the most frequently performed [30, 32]. The rate of post-operative complications ranged from 8 to 35%. No mortality was recorded following surgery in any of the 3 RCTs.

**Table 3:** Summary of endoscopic treatment

	Díte et al. [29] (*n* = 64)	Cahen et al. [30] (*n* = 19)	Issa et al. [32] (*n* = 44)
Number of endoscopic procedures — median (IQR)	2* (1–4)	5 (1–11)^$^	3 (1–4)
Presence of ductal stones — No. (%)	NS	18 (95)	29 (74)
Presence of ductal stricture — No. (%)	NS	Stricture alone 1 (5) Stricture and stones 16 (84)	Stricture and stones 34 (77)
Use of lithotripsy — No. (%)	NS	16/18 (89)	22/29 (76)
Number of lithotripsy sessions	NS	1 session (10 patients) Multiple sessions (6 patients)	Median 1 session (IQR 0–1)
Number of patients who were stented — No./total (%)	33/63 (52)	16/19 (84)	29/39 (74)
Number of patients requiring balloon dilatation — No./total (%)	NS	15/16 (94)	32/39 (82)
Complete duct clearance — No./total (%)	NS	16/18 (89)	24/39 (62)
Complications — No. (%)	5 (8) Bleeding — 2 cases Acute pancreatitis — 2 cases Pancreatic abscess — 1 case	11 (58) Skin wound post-lithotripsy — 1 case Stent related complications — 5 cases Pancreatitis — 4 cases Cholecystitis — 1 case	11 (25)
Mortality — No. (%)	0 (0)	1 (5%) Perforated duodenal ulcer flowing lithotripsy	0 (0)

*NS* not specified. *Presented as mean, ^$^presented as range

**Table 4:** Summary of surgical treatment

	Díte et al. [29] (*n* = 76)	Cahen et al. [30] (*n* = 20)	Issa et al. [32] (*n* = 41)
Surgical management			
Resection — No. (%)	61 (80) Duodenum-preserving pancreatic head resections — 33 Hemipancreatoduodenectomy — 23 Distal pancreatectomy — 5	1 (5) Whipple procedure — 1	17 (41) Duodenum-preserving pancreatic head resection — 15 Pylorus-preserving pancreatoduodenectomy — 1 Distal pancreatectomy — 1
Drainage procedure — No. (%)	15 (20) Partington-Rochelle — 15	18 (90) Pancreaticojejunostomy — 18	24 (59) Lateral pancreatojejunostomy — 24
Resection + drainage procedure — No. (%)		1 (5) Frey procedure — 1	
Complications — No. (%)	6 (8) Acute pancreatitis — 2 cases Fistula — 2 cases Ileus — 1 case Anastomotic leak — 1 cases	7 (35) Pneumonia — 1 case Wound infection — 3 cases Bleeding — 2 cases Anastomotic leak — 1 case	12 (29)* Anastomotic leak — 3 cases Bleeding — 3 cases Incisional hernia — 2 cases Pneumonia — 2 Severe delayed gastric emptying — 2 cases Sepsis — 1 case
	Repeat surgery 2 (3) Ileus — 1 case Anastomotic leak — 1 case	Repeat surgery 1 (5) Anastomotic leak — 1 case	Repeat surgery 3 (7) Bleeding — 2 Diagnostic — 1
Mortality — No. (%)	0 (0)	0 (0)	0 (0)

^*^More than 1 complication per patient reported

The risk of bias for each individual study was evaluated (supplementary Fig. 1). No study received a low risk of bias for all domains assessed. Due to the nature of the treatment strategies proposed, patient blinding is not feasible. No information was recorded within the study of Díte et al. with regard to any additional attempts to introduce some form of blinding to the trial personnel within the study [29]. Cahen et al. described that patients completed their questionnaires in private and that only the study coordinator had access to clinical records whereas Issa et al. did blind the statistician for the analysis of the primary outcome measure [30, 32]. A lack of patient blinding could impact on the study endpoint, notably with the subjective nature of reporting of pain. Preconceived conceptions by patients regarding the superiority of either treatment could introduce a bias to the results, whereas a lack of blinding would not affect secondary outcome measures such as exocrine or endocrine functions. A high risk of bias was noted in the trial by Díte et al. [29]. Limitations in the trial design included the use of pseudo-randomisation and unconcealed allocation. Recorded patient demographics with important confounding factors such as concurrent alcohol use and active smoking status were also missing. Patients with an enlarged pancreatic head were included; however, it was unclear whether stratification for this feature was performed.

A meta-analysis was performed with the available data for patient outcomes following surgical or endoscopic management of their CP (Table 5). Cahen et al.’s [31] study was used as this contained the results of a 5-year follow-up in order to determine whether outcomes were sustained over time [31]. Three specific areas of patient outcomes were analysed: pain control, quality of life and functional outcomes. For pain control, an initial analysis of any pain relief post-procedure was conducted. Pain control was defined by changes in the Melzack score by Díte et al., whereas both Cahen et al. and Issa et al. utilised the Izbicki pain score [29, 30, 32]. A significantly higher rate of any pain relief was noted following surgery, in 100/131(76%) patients who underwent surgical management in comparison to 64/121 (53%) patients who received endoscopic therapy [OR (95% CI) 2.91 (1.65–5.12), *p* = 0.0002] with low heterogeneity (*I*^2^ = 0%) (Table 5). Subgroup analyses were performed to quantify the extent of pain control. A significantly higher rate of complete pain control was achieved following surgery (48/131 (37%) patients) when compared to endoscopy (21/121 (17%) patients) [OR (95% CI) 2.79 (1.53–5.08), *p* = 0.0008] with low heterogeneity (*I*^2^ = 0%). No difference was noted between either intervention for partial pain control [OR (95% CI) 1.13 (0.66–1.93), *p* = 0.66] with low heterogeneity (*I*^2^ = 0%). A significantly higher rate of no pain relief was noted in CP patients following endoscopy (57/121 (47%) patients) when compared to surgical management (30/131 (23%) patients) [OR (95% CI) 0.33 (0.18–0.58), *p* = 0.0001] with low heterogeneity (*I*^2^ = 0%). This finding was reproduced when assessing the post-treatment Izbicki score following intervention. A significantly higher Izbicki score was noted in patients treated in the endoscopy arm at follow-up [MD (95% CI) − 13 (− 22.34 to 3.66), *p* = 0.67] with low heterogeneity (*I*^2^ = 0%). All three trials have a relatively small sample size. The trial by Díte et al. presents both a small randomised cohort of patients (endoscopic therapy 36 patients and surgery 36 patients) and a larger total group that encompasses both randomised and non-randomised patients [29]. We used and analysed the total group of patients as this would ensure adequate numbers and sufficient power for the analysis. We subsequently performed a subgroup analysis of only the randomised patients from Díte et al. and assessed pain control outcomes (supplementary material) [29]. The findings were consistent with our previous results. A significantly higher rate of complete pain control was achieved following surgical management (32/91 (35%) patients) when compared to endoscopic therapy (17/93 (18%) patients) [OR (95% CI) 0.41 (0.20–0.81), *p* = 0.01]. Higher rates of no pain relief occurred following endoscopic therapy (24/52 (46%) patients) when compared to surgical management (8/51 (16%) patients) [OR (95% CI) 4.71(1.84–12.09), *p* = 0.001] (Table 5).

**Table 5:**
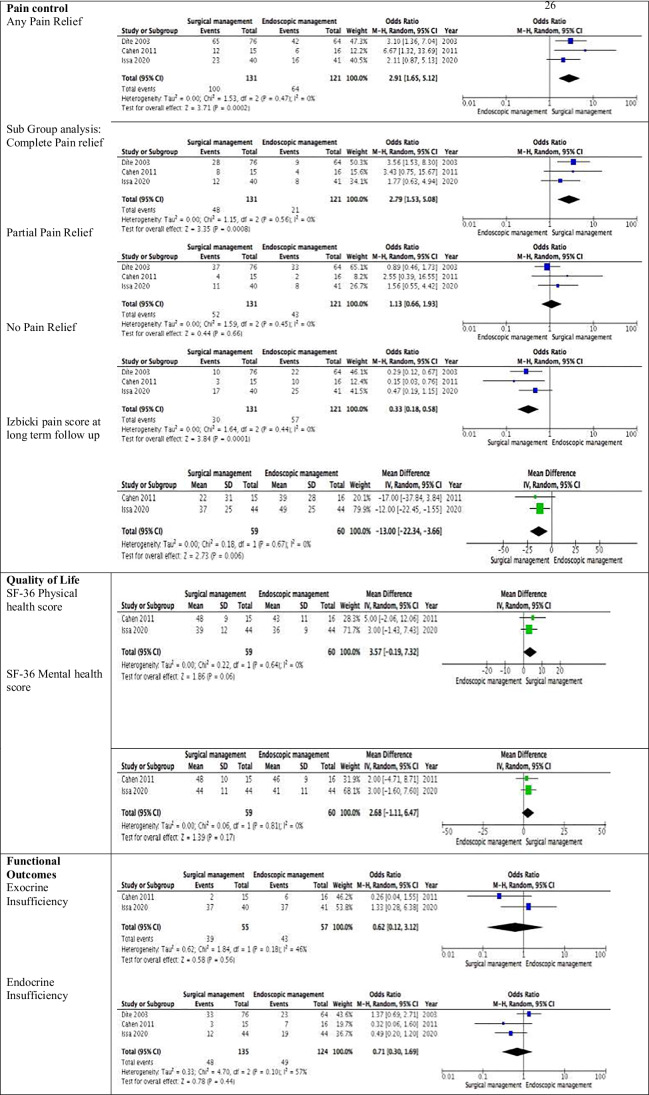
Meta-analysis of primary and secondary outcomes

The impact of surgery or endoscopy on quality of life was assessed by the results of the SF-36 questionnaire. The result of the questionnaire is on a scale that ranges from 0 (worst health) to a 100 (best health). For the effect on physical health, a trend towards surgery improving the physical health score was observed; however, this was not statistically significant (*p* = 0.06) [MD (95% CI) 3.57 (− 0.19 to 7.32)]. No significant difference was noted in the SF-36 mental health score following either intervention [MD (95% CI) 2.68 (− 1.11 to 6.47), *p* = 0.17].

In order to evaluate functional outcomes, the rates of new onset exocrine and endocrine failure following surgery and endoscopy were assessed. Of the two trials that reported on exocrine insufficiency, faecal elastase was utilised as a diagnostic test [30, 32]. The definition of endocrine failure varied; Díte et al. reported new onset diabetes during follow-up (with no reference to a measurable diagnostic test), Cahen et al. defined endocrine insufficiency as the need for hyperglycaemic treatment due to a raised fasting glucose or glycated haemoglobin level, and Issa et al. also used the need for hyperglycaemic treatment as their definition [29, 30, 32]. No statistical difference was noted in exocrine failure rates between surgical management and endoscopic therapy (39/55 (71%) of patients following surgery and 43/57 (75% of patients following endoscopic therapy)) [OR (95% CI) 0.62 (0.12–3.12), *p* = 0.56]. No difference was noted in endocrine failure rates following surgical management (48/135 (36%) patients) or endoscopic therapy (49/124 (40%) patients) [OR (95% CI) 0.71 (0.30–1.69), *p* = 0.44].

## Discussion

This meta-analysis highlights that when compared to endoscopic therapy, surgery is associated with significantly higher rates of complete pain control at long-term follow-up (37% of patients following surgical management and 17% of patients following endoscopic therapy). Furthermore, the outcome of the trial by Issa et al. demonstrates that early surgery (within 6 weeks of randomisation) resulted in lower pain scores at 18-month follow-up in comparison to the endoscopy first approach [32]. From the current literature, it is known that approximately 40–75% of patients with CP will require surgery [33]. Escalating through a progressive stepwise management plan may introduce a time lag delay, where definitive treatment is postponed. Early surgery may also be associated with several other theoretical benefits. A significant proportion of patients with CP will be using opiate analgesia in an attempt to manage their pain. Terrace et al. demonstrated that higher rates of absolute pain relief can be achieved following surgery if the patient is opiate naïve perioperatively [34]. This finding was further echoed in a published systematic review in 2014 [35]. The exact mechanism of dysregulated pain sensation in CP has yet to be understood. Persistent localised inflammatory changes may contribute to pancreatic neuropathy [36]. Concurrent and prolonged opiate use may result in dysfunctional pain transmission between the peripheral and central nervous system, thus resulting in central sensitisation, a phenomenon defined by hyperalgesia and allodynia (excessive pain response from a minimal painful stimulus or from a non-harmful stimulus) [37]. In addition, long-term opiate use may result in narcotic bowel syndrome, a condition of bowel dysfunction that is defined by chronic abdominal pain that worsens with increasing doses of analgesia [20]. One may argue that early surgery would reduce opiate usage and dependency rates, which may have a positive impact on long-term outcomes for pain control. Another potential benefit of early surgery is that progressive fibrosis of the pancreatic parenchyma secondary to chronic inflammation would be halted; thus, preservation of functioning islet cells and an intact acinar compartment may permit long-term competency of both pancreatic exocrine and endocrine function [38]. Due to the subjective nature of pain, its use as an outcome measure can only be used with a standardised reporting tool (such as the Izbicki pain score). However, it is important to consider as to what defines a meaningful reduction in the pain score. Both Díte et al. and Issa et al. provided predefined numeric values (pain score reduction value) of what constituted a therapeutic improvement in pain control [29, 32]. The use of different thresholds can impact the results and introduce a bias.

Several different surgical approaches towards managing CP have been described over the decades. The definitive approach has yet to be determined. Heterogeneity of surgical practice is noted in this meta-analysis. A published network meta-analysis in 2020 of surgical strategies for CP demonstrated that the Beger procedure had the best ranking score for pain relief [39]. However, the Frey procedure scored greater for postoperative quality of life and exocrine insufficiency [39]. The surgical intervention should be guided by the distribution of the disease, whether there is an inflammatory mass present within the pancreatic head or if there is widespread main duct disease with dilatation of the pancreatic ductal system. The previously reported high failure rates of conventional drainage procedures have raised a suspicion that the pancreatic head may serve as the main pacemaker region for pain [36, 40]. Patients with CP with pancreatic head involvement have different symptomology and a clinically different disease course when compared to individuals with disease predominantly in the body or tail (41). Regardless as to whether pancreatic head enlargement is present, surgical management is superior to endoscopic therapy. Published case series of total pancreatectomy for CP have also demonstrated a significant improvement in pain control and reduction in post-procedure opiate use at long-term follow-up [41–43]. Whilst discussing surgical treatment options for CP, it is important to acknowledge the results of the ChroPac trial [44]. This large, multicentre, double-blind superiority trial evaluated the efficacy of duodenum-preserving pancreatic head resection (DPPHR) and partial pancreatoduodenectomy for CP [44]. At long-term follow-up, both surgical approaches had comparable results with regard to pain control, functional outcomes and quality of life [44]. This illustrates the extent of symptomatic control that can be achieved with surgery and that both these procedures can serve as a reference standard for treatment.

Another important discussion point is the frequency of treatment. Whilst surgery is definitive and provides treatment at a single time point within a hospital admission, this differs significantly to endoscopic therapy. Endoscopic management of CP will require serial interventions. The time course of treatment is prolonged and may significantly impact on the patient. Numerous hospital visits may have a detrimental effect on education or employment. Treatment for CP should allow for social re-integration rather than further isolation.

A similar meta-analysis comparing endoscopic therapy and surgery for CP was published by Mendieta et al.; however, this meta-analysis did not assess endocrine/exocrine function or quality of life as treatment outcomes [45]. Quality of life is an important metric to be studied in this cohort of patients with CP. Due to the relapsing nature of the disease, there is a significant psychosocial impact. This is reflected in a study by Gardner et al.; a survey conducted of patients with CP illustrated high rates of unemployment (27%), impairment in performing daily activities and high rates of negative health care experience [46]. As a consequence, societal segregation and marginalisation may occur. Within this meta-analysis, a trend towards surgery improving the physical health score at follow-up was observed albeit not statistically significant. This may be reflective of the fact that surgery was associated with higher rates of complete pain control and that this impacts on the domains assessed in the SF-36 questionnaire. However, what is needed is long-term follow-up data regarding social functioning, specifically whether re-integration into society occurs and successful continuous employment. This highlights the need for a comprehensive multidisciplinary team approach (including social workers, specialist pain teams and counsellors) in managing CP patients in both the perioperative and post-hospital discharge setting.

When investigating long-term functional outcomes, this meta-analysis failed to demonstrate a significant difference between new onset exocrine or endocrine failure following surgery or endoscopic management. However, the limitations of the included trials should be considered. Closer interrogation of the data demonstrates that 2 of the 3 included RCTs displayed higher rates of new onset endocrine failure following endoscopy [30, 32]. However, Díte et al. presented higher rates of endocrine failure following surgery [29]. It is important to highlight that Díte et al. adopted a much more aggressive surgical approach to managing CP (80% underwent resection) [29]. In both other RCTs, a drainage procedure was most frequently performed (80% and 55% respectively). It is possible that the extended pancreatic resection in combination with the residual fibrotic non-functioning parenchyma resulted in the higher rates of endocrine failure following surgery in Díte et al. [29]. This should be taken into consideration when assessing the long-term functional outcome data. However, there is an important factor missing within these RCTs and this meta-analysis, that is, the use of total pancreatectomy with islet auto-transplantation (TPIAT) for CP management. Published case series of TPIAT have demonstrated high rates of narcotic independence at long-term follow-up [47]. The use of islet auto-transplantation serves as an opportunity to preserve β-cell function in an attempt to maintain endocrine function. Insulin independence post-TPIAT varies across published series [47–51]. The islet cell yield and successful engraftment of the transplanted islets are two essential factors that influence long-term endocrine function. For patients with longstanding CP or with frequent acute attacks, atrophic and fibrotic parenchymal changes throughout the pancreas with or without calcification may occur. These features have been associated with a reduced yield of functioning islets for auto-transplantation [47–51]. Prior pancreatic surgery has a detrimental effect on the islet cell yield [47, 51]. In light of the aforementioned aspects (the negative impact of disease chronicity prior to intervention on the gland and the impact of previous surgery on islet cell yield), one could propose that early intervention with TP-IAT would allow not only symptomatic relief but also a greater chance of long-term endocrine function.

There are limitations to note within this meta-analysis. Long-term follow-up varied between the included RCTs, of which 2 reported 5-year follow-up data [29, 31]. The remaining RCT published 18 months of follow-up [32]. For assessing the impact of treatment on CP, it is essential that outcomes are assessed longitudinally over time in order to determine whether the effect of surgery or endoscopy is sustained. The lack of published patient demographics in Díte et al. makes an assessment and comparison of the patient cohort with the other studies difficult [29]. Heterogeneity amongst the patient cohorts between studies was noted in addition to the variation in the timing of surgery. Whilst assessing long-term outcomes following surgery or endoscopy for CP, some important variables are missing: notably quantifying the usage of opiates/narcotic independency rates, re-employment rates and social functioning measures. To assess long-term endocrine function, further quantifiable data is required such as c-peptide results, HbA1c and insulin requirements. This raises the question as to whether standardised data outcome set for determining the effect of an intervention on CP is required.

## Conclusion

Surgical management is associated with superior pain control when compared to endoscopic therapy for CP and should therefore be considered first-line treatment. The evidence generated within this meta-analysis illustrates that significantly higher rates of complete pain control were achieved following surgery during long-term follow-up. A trend towards surgery improving physical health was observed. No statistical difference was noted between the rates of new onset exocrine or endocrine failure following either treatment strategy. Further research is required to determine the impact of early up-front TPIAT in patients with CP in order to assess whether prompt intervention following diagnosis can secure long-term endocrine function.

## Supplementary Information

Below is the link to the electronic supplementary material.

Supplementary file1 (DOCX 2255 KB)

Supplementary file2 (DOC 64 KB)

## Data Availability

All data is readily available within the previously published articles. This manuscript is a systematic review and meta-analysis of the concurrent literature.

## Code availability

No specific coding was required for this meta-analysis. The data was analysed using the Review Manager software.
