# Supplementary material for: A meta-analysis of the long-term outcomes following surgery or endoscopic therapy for chronic pancreatitis
Source: Langenbecks Arch Surg. 2022 Mar 22;407(6):2233–45. doi: 10.1007/s00423-022-02468-x (PMC9468079; doi:10.1007/s00423-022-02468-x)
Supplement: Supplementary file 1 — Supplementary file1 (DOCX 2255 KB) [file 423_2022_2468_MOESM1_ESM.docx]

**Supplementary material**

Supplementary table 1 – search criteria

| Domain | Search criteria |
| --- | --- |
| Patients | Chronic Pancreatitis  Familial Pancreatitis  Hereditary Pancreatitis  RCT |
| Intervention | Treatment  Management  Surgery  Resection  Frey’s procedure  Partington Rochelle  Puestow procedure  Bergers  Whipple |
| Comparator | Treatment  Management  Endoscopic  Stent  Drainage  Sphincterotomy  Stone extraction  Mechanical lithotripsy  Extracorporeal shock-wave lithotripsy |
| Outcomes | Pain control  Quality of life  Endocrine function  Exocrine function. |

Supplementary table 2 – Reported outcome measures and definitions within each study

| Outcomes | Díte et al ^29^ | Cahen et al ^30^ | Issa et al ^32^ |
| --- | --- | --- | --- |
| Primary outcome measure  Description | Pain control  Complete response defined as an absence of pain or attacks of pancreatitis pain  Partial response defined as a reduction in pain levels by a score of three points on the Melzack score  No response defined by no pain improvement or similar episodes of pain attacks or worsening of symptoms | Pain control  Complete pain relief was defined as at the end of follow up as a Izbicki pain score ≤10  Partial pain relief was defined as at the end of follow up as a Izbicki pain score >10 after a decrease of  >50% | Pain control  Complete pain relief at the end of follow up was defined as Izbicki pain score ≤10  Partial pain relief at the end of follow up was defined as Izbicki pain score >10 but more than 50% decrease compared with the baseline score] |
| Treatment failure  Description | Need for further treatment interventions (Both endoscopic and surgery) | Conversion of treatment strategy from endoscopic to surgery or vice versa. Death secondary to treatment | Failure of medical therapy was defined by a persistent VAS sore of 4 for 6 weeks. Failure of Endoscopic treatment was defined by a persistent VAS sore of 4 for 6 weeks or if stenting was still required after 1 year for symptomatic control |
| Secondary outcome measure  Endocrine failure  Description  Exocrine failure  Description  Quality of life  Definition | New onset diabetes during follow up. No reference to a specific diagnostic test  Not assessed  Not assessed | Requirement for hyperglycaemic medication. Treatment instigated if fasting glucose >6.7 mmol/L or glycated heamoglobon >6%  Fecal elastase  SF-36 questionnaire | Endocrine insufficiency defined by the use of diabetes medication. Treatment advised if glycated heamoglobon >7%  Fecal elastase  SF-36 questionnaire |

Supplementary table 3 - GRADE review of evidence quality

| A Meta-analysis of the long-term outcomes following endoscopic therapy or surgical management for Chronic Pancreatitis | | | | | | | | | |
| --- | --- | --- | --- | --- | --- | --- | --- | --- | --- |
| Summary of findings | | | | Quality assessment | | | | | |
| Outcome of interest | Number of patients (studies) | Pooled Effect Size (95% CI) | Anticipated Effects | Risk of Bias | Inconsistency | Indirectness | Imprecision | Publication Bias | Overall Certainty of Evidence (GRADE) |
| Complete pain control | 252  (3) | OR 2.79  95% CI = 1.53 - 5.08, P=0.0008,  I^2^ = 0% | Significantly higher rate of complete pain control following surgical management | Not serious * | Not serious | Not serious | Moderate^^@^ | Not serious | High |
| No pain relief | 252  (3) | OR 0.33  95% CI = 0.18 – 0.58,  P = 0.0001,  I^2^ = 0% | Significantly higher rate of no pain control following endoscopic management | Not serious * | Not serious | Not serious | Moderate^^@^ | Not serious | High |
| SF-36 Physical health score | 119  (2) | MD 3.57  95% CI =-0.19 – 7.32  P = 0.06  I^2^ = 0% | No difference between either treatment strategy | Not serious * | Not serious | Not serious | Serious ^#^ | Not serious | Low |
| SF-36 Mental health score | 119  (2) | MD 2.68  95% CI = -1.11 – 6.47  P = 0.17  I^2^ = 0% | No difference between either treatment strategy | Not serious * | Not serious | Not serious | Serious ^#^ | Not serious | Low |
| Exocrine insufficiency | 112  (2) | OR 0.62  95% CI= 0.12 – 3.12,  P = 0.56  I^2^ = 46% | No difference between either treatment strategy | Not serious * | Serious ^£&^ | Not serious | Serious ^#/^ | Not serious | Low |
| Endocrine  insufficiency | 259  (3) | OR 0.71  95% CI = 0.30 – 1.69,  P = 0.44  I^2^ = 57% | No difference between either treatment strategy | Not serious * | Serious ^£&^ | Not serious | Serious ^#/^ | Not serious | Low |
| * Articles included in this meta-analysis were RCTs. However, in light of the proposed treatment algorithms patient blinding was not possible. Two of the included studies did attempt to introduce some form of blinded assessment into the analysis  ^ Reduced patient sample size at long term follow up in the study by Cahen et al  @ overall small cohort of patients for meta-analysis  £ moderate degree of heterogeneity I^2^ >40%,  # low number of studies and patients available for meta-analysis  / Effects favouring both adjuvant therapy and observation alone noted, suggestive of the need for more studies with larger sample sizes  & Little overlap of confidence intervals | | | | | | | | | |
| RCTs – Randomised Control Trials, OR – Odds Ratio, MD – Mean difference, CI – Confidence interval | | | | | | | | | |

Supplementary figure 1 – Meta analysis of pain outcomes following surgery or endoscopic therapy. Subgroup analysis of only the randomised patients from the study of Dite et al 2003

Complete pain control


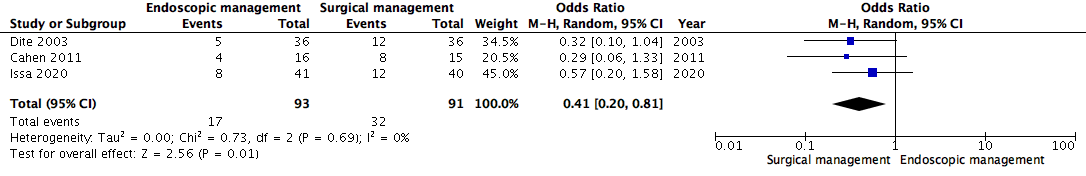


Partial pain control


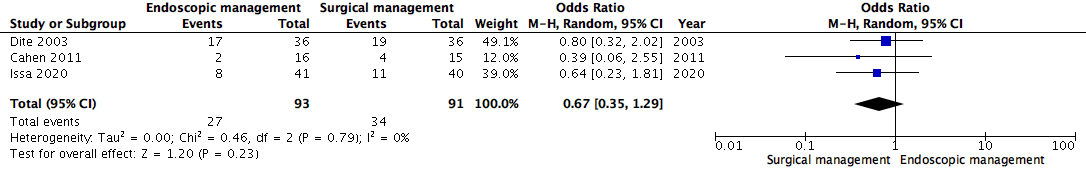


No pain relief


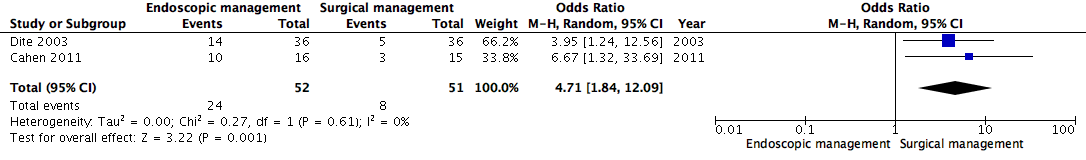


Supplementary figure 2 – Risk of Bias assessment with ROB2


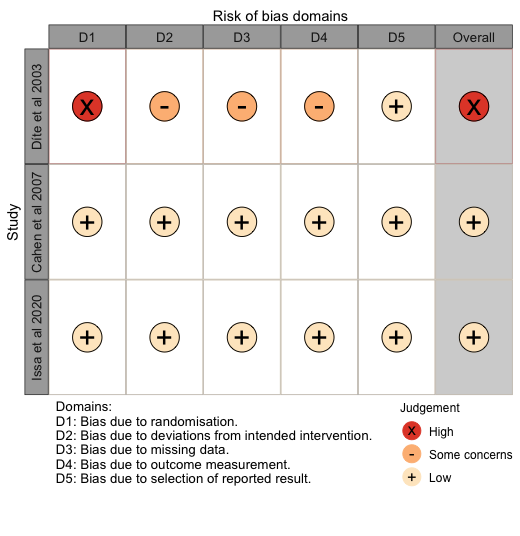


=
